# Application Fields, Positions, and Bioinformatic Mining of Non-active Sites: A Mini-Review

**DOI:** 10.3389/fchem.2021.661008

**Published:** 2021-05-31

**Authors:** Xiaoxiao Wang, Qinyuan Ma, Jian Shen, Bin Wang, Xiuzhen Gao, Liming Zhao

**Affiliations:** ^1^ School of Life Science, Shandong University of Technology, Zibo, China; ^2^ Shandong Jincheng Pharmaceutical Group Co.LTD, Zibo, China; ^3^ State Key Laboratory of Bioreactor Engineering, East China University of Science and Technology, Shanghai, China; ^4^ Research and Development Center of Separation and Extraction Technology in Fermentation Industry, East China University of Science and Technology, Shanghai, China

**Keywords:** non-active sites, enzyme engineering, biocatalyst, bioinformatics mining, molecular mechanisms

## Abstract

Active sites of enzymes play a vital role in catalysis, and researchhas been focused on the interactions between active sites and substrates to understand the biocatalytic process. However, the active sites distal to the catalytic cavity also participate in catalysis by maintaining the catalytic conformations. Therefore, some researchers have begun to investigate the roles of non-active sites in proteins, especially for enzyme families with different functions. In this mini-review, we focused on recent progress in research on non-active sites of enzymes. First, we outlined two major research methodswith non-active sites as direct targets, including understanding enzymatic mechanisms and enzyme engineering. Second, we classified the positions of reported non-active sites in enzyme structures and studied the molecular mechanisms underlying their functions, according to the literature on non-active sites. Finally, we summarized the results of bioinformatic analysisof mining non-active sites as targets for protein engineering.

## Introduction

Enzymes are used widely in industrial production, energy development, medical and health industries, and life science researchas biocatalysts. Each enzyme molecule has active sites related to its function. These active sites are usually located deep in the catalytic cavity. The substrate gets attached to the active site and binds to the amino acids in the active pocket through different interactive forces, such as hydrogen bonds, ionic bonds, and van der Waals force interactions. Mutations in amino acid residues in many active sites, especially mutations in key amino acid residues related to the activity of the enzyme, have a great impact on the properties of the enzyme, indicating that the active site of the enzyme is very important for enzymatic reactions. For example, ribulose-1,5-bisphosphate carboxylase-oxygenasecatalyzes the fixation of CO_2_ to form an organic acid. Poudel verified through computational approaches that regions around the active sites of the enzyme play key roles in the discrimination of CO_2_ and O_2_ binding to the enzyme (Poudel et al., 2020). Therefore, in order to understand the relationship between the structure and function of enzymes and to change the catalytic performance of enzymes, active sites are often the preferred mutation sites for protein engineering.

However, in some functionally different enzyme families, studying the active site alone cannot comprehensively explain the catalytic mechanism of the entire enzyme family. For example, New Delhi metallo-β-lactamase-1 (NDM-1) is a carbapenemase that can hydrolyze almost all *ß*-lactamase antibiotics. To date, twenty-one variants of this enzyme have been confirmed. Previous studies have revealed the presence of active site residues (Kim et al., 2013). NDM-1 showed low sequence identity with all its variants compared to other *ß*-lactamasesand displayed rearrangements around the active site (King and Strynadka, 2011). Therefore, amino acid residues around the active sites are structurally and functionally related (Ali et al., 2018; Ali et al., 2019).

In this mini-review, we focused on the progress in research on non-active sites and summarized their application fields, locations in the enzyme structure, molecular mechanisms, and bioinformaticmining methods.

### Application Fields of Non-active Sites

In recent years, an increasing number of scientists have shifted the focus of protein engineering from active sites to peripheral or remote non-active sites outside the active site. In this section, we summarize two major approaches tothe current research on non-active sites.

### Understanding Catalytic Mechanisms Through Non-active Sites

Table 1 showsthe research approaches to the understanding of catalytic mechanisms through non-active sites. For example, GH26-type endo-β-1,4-mannanase CrMan26 exhibited a preference for galactomannan as a substrate and a unique orientation of the ancillary domain. By sequence alignment of CrMan26 with other endo-β-1,4-mannanases from the GH26family, Mandelli et al. targeted the distal Tyr195 residue (Mandelli et al., 2020). Tyr195 was shown to be involved in substrate preference by mutation studies (Tyr to Ala mutation) and subsequent kinetic characterizations. Crystal structures indicated that the distal negative residue Tyr195 cooperated with the ancillary domain.

**TABLE 1:** Research on understanding catalytic mechanisms of non-active sites.

Enzymes	Characteristics	Non-active site mutation	Reference
GH26-type endo-β-1,4-mannanase (CrMan26)	Preference for galactomannan as a substrate	Y195A	Mandelli et al. (2020)
New Delhi metallo-β-lactamase (NDM-1)	21 different NDM variants	K125A, D192A, D122A, Trp93A	Khan and Rehman (2016); Ali et al. (2019)
O-GlcNAc transferase	Substrate specificity	D386A, D420A, D454A	Joiner and Levine (2019)
*Meso*-diaminopimelate dehydrogenase (StDAPDH)	Substrate specificity	R71A/E/K/Q/S/I	Zhang et al. (2018)
Human DNA polymerase *β*	Key role in the phenotypic result	S229L, G231D	Eckenroth et al. (2017)
Exo-inulinase	Hydrolysis of inulin	H192A	Arjomand et al. (2017)

### Enzyme Engineering for Improved Performance With Non-active Sites as Targets

As stated above, non-active sites may be targeted for directed evolution or rational design toimprove the performance of an enzyme. For example, in order to engineer transketolase (TK) to accept an unnatural donor (pyruvate) and acceptor (aliphatic or aromatic aldehydes), Yu et al.performedstructural alignment between TK and pyruvate decarboxylase (PDC). Furthermore, amino acid residues of TK, those around the substrate of PDC, were selected for mutagenesis (Yu et al., 2020). Thus, non-active sites could be considered good candidates for improving enzymeperformance.

#### Increase in Catalytic Efficiency

Usingendo-polygalacturonase PG8fn as a template, the non-active site residue Thr113located in the T3 loop of the enzyme, was targeted to create nine mutants based on the characteristics of amino acids, including polarity, charge, and sterics (Tu et al., 2016). The best variant T113R showed an approximately 50% increase in catalytic efficiency, and most non-active sites that were verified to be beneficial to catalytic efficiency were obtained by directed evolution, such as random mutagenesis and DNA shuffling, and not by site-directed mutagenesis with non-active sites as targets.

#### Alteration of Substrate Scope

The narrow substrate scope of some enzymes is a major obstacle in the application of biocatalysts. To expand the scope of threoninedeaminase (TD) to catalyzebulky substrates, Song et al.designed experiments and found that the TD from *Corynebacterium glutamicum*could accommodate a bulky substrate such as phenylserine. They focused on the amino acids located in the substrate tunnel, including the gate constituent residues, anchoring residues, and hinge residues (Song et al., 2020). By the first round of alanine scanning, eight key residues whose activities were ≥30% higher than the wild type(WT) were screened; subsequently, three mutation libraries were constructed by saturation mutation and iterative mutation. Finally, a combinational variant CgTDMu7 was constructed based on the best mutants from the second round of mutagenesis, whichexhibited a 6.8-fold higher activity.

#### Improvement of Thermostability

The thermostability of enzymes is a very important factor for their successful industrial application because of the elevated temperatures during industrial processes. Acombination of several forces such as hydrophobic interactions, salt bridges, disulfide bonds, and hydrogen bonds, lead to decreased flexibility of enzymes. However, there is a negative correlation between enzyme activity and stability during industrial applications and directed evolution (Yu and Huang, 2014). Because of this trade-off, amino acid residues located in flexible regions of the enzyme, such as loop regions, are usually targeted for mutagenesis during protein engineering to improve the thermostability of enzymes (Ning et al., 2018; Zhang et al., 2019). For example, *Escherichia coli* transketolase (TK) 3M was a variant obtained by saturation mutagenesis and showed a 9.6-fold higher activity relative to the WT enzyme. To increase its thermostability, Yu and Dalby shiftedtheir focus to the residues His192, Ala282, Ile365, and Gly506, which are located in the dimer-interface regions (Yu and Dalby, 2018a). After mutations, the best variant, 7M, showed a 10.8-fold improved half-life at 55°C.

## Positions of Reported Non-active Sites in the Structures of Enzymes

Although some studies have focused on non-active sites for many enzymes, the role of non-active sites in enzymatic reactions has not been investigated. The majority of thenon-active sites reported todateare located in the flexible loop regions. According to the functions of the loop region, the positions of the nonactive sites can be divided as follows:

### Substrate Tunnel

For substrate catalysis, the prerequisites are as follows: 1) the substrate should fit into the binding pocket of the enzyme, and 2) the substrate should pass through the tunnel (Song et al., 2020). In most enzymes, the active site is located in the internal cavity, while the tunnels are connections between the active sites and the solvent (Kokkonen et al., 2019). Therefore, non-active sites located in tunnels are an alternative approach tounderstanding catalytic mechanisms and enzyme engineering (Song et al., 2020). In the tunnel, 47 non-conserved amino acid residues were located; this included 18 gate constituent residues, 12 anchoring residues, and 17 hinge residues which were selected for the expansion of the substrate scope of the enzyme (Song et al., 2020). Such an approach has also been applied in other studies (Zhou et al., 2013).

### Protein Surface

Amino acid residues located on the surface of the enzyme may play critical roles in the performance of the enzyme, and may be located either on the surface of the enzyme or on the dimer interface (Taylor et al., 2015; Yu and Dalby, 2018b). For example, in the best variant of dialkylglycine decarboxylase (DGD) with altered substrate specificity, Asn203, Asn96, Arg85, and Asn12 were near the surface of the enzyme. Asn203 is located near the dimer-dimer interface, Asn12 islocated in the N-terminal *a*-helix and within the interactive distance of residue Arg85, and Asn96 was positioned at either end of the *a*-helix above Asn306 (Taylor et al., 2015).

### Loop Region

The amino acid residue Lys253 of the arylsulfatase enzyme from *Pseudoalteromonascarrageenovora*islocated in the loop region of the enzyme (Zhu et al., 2020). It was found that the mutation of Lys253 to Glnduring directed evolution increased arylsulfatase activity when compared to the WTenzyme. Protein structure analysis indicated that the loop which contains residue Lys253 acts neither as a tunnel nor as an interactivesurface for proteins and was a common loop. K253Q showed increased interactionswith the substrate than the WT enzyme by forming a higher number of hydrogen bonds with its adjacent amino acids. The amino acid residue Tyr55 of mammalian d-amino acid oxidases is located in the loop around the enzyme active sites (Kalyanasundaram et al., 2018). It was found that mutation of Tyr55 improved the enzyme characteristics. Molecular dynamics simulations (MD) analysis indicated that the Tyr55 residue could vary substrate specificity to a greater extent, which is attractive for industrial applications (Kalyanasundaram et al., 2018).

## Molecular Mechanisms of Non-active Sites in Enzymes

The substitutions of single amino acid residuesinthe side chains of amino acid residuestend to have minor local consequences on the overall structure of the protein. Therefore, the molecular mechanisms underlyingthe effect of mutagenesis of non-active sites on enzyme function include conformational changes induced by interactions within the protein. This can be manifested in the following ways.

### Mutagenesis Induces Conformational Changes in Catalytic Pockets

Liu et al. found that during the evolution of GH11 endoxylanase, the substitution of distal residues N29S, S31R, and I51V did not alter the tertiary structure of the WT enzyme. However, slight changes were inducedin the active site architecture by weakening of the interactions between “gate” residues and active sites (Liu et al., 2019). Finally, the conformation of variants DS241 and DS428 changed to an “open state,” which improved their activity toward xylan. Similar changes were observed in the DGD. Mutations in the non-active sites opened the active sites by the movement of small domains on the surface of the enzyme, which made the variant accept larger substrates (Taylor et al., 2015). Li et al. found that the Leu343R mutation of glycogen synthase kinase 3β, located at the C-lobe which is remote from the catalytic site, disrupted the hydrophobic environment formed by residues Leu160, Tyr163, and Phe340, and affected the conformational rearrangement of the activation loop (A-loop). The conformational rearrangement of the A-loop disrupted the primed phosphate binding site, which abrogated the catalytic activity of glycogen synthase kinase 3β (Li et al., 2019).

### Mutagenesis Induces Conformational Changes in Substrate Tunnels

As stated above, some non-active sites are located in theenzyme tunnel; therefore, mutations at these sites induce conformational changes in the tunnel (Tu et al., 2016; Song et al., 2020). For example, by engineering the residues located in the tunnel of CgTD, mutations of the hinge residues Val119, Lys123, and Val137 cause a kink in the loop from His132 to Asn144, which subsequently changes the state of the tunnel from open to closed (Song et al., 2020). The amino acid residue Tyr55 of humand-amino acid oxidases (hDAAO) is located in the loop around the enzyme active sites. The mutation at this position showed increased varied substrate specificity and improved enzyme characteristics (Kalyanasundaram et al., 2018). Tunnel analysis indicated that there were three tunnels in hDAAO, and Tyr55 was one of the residues that separated the T1 tunnel from the T2 tunnel. Tyr55 was important in regulating the properties of T1, T2, and T3 tunnels, which were calculated using CAVER (Kalyanasundaram et al., 2018).

## Bioinformatic Tools for the Mining of Non-active Sites for Protein Engineering

Through random mutagenesis and/or directed evolution, such as protein engineering of mammalian cytochrome P450 2B1, enzyme variants with improved enzymatic characteristics have been obtained, in which non-active sites may be involved (Kumar et al., 2005). However, for direct targeting of non-active sites by protein engineering,a pre-assay is necessary to select the target position. In this section, we summarize some bioinformatic tools for miningnon-active sites.

### Multiple-Sequence Alignment Based on Primary Structure

It is possible to find the difference in amino acid types in the primary structure of enzymes by comparing members of an enzyme family with different catalytic properties or low similarity. The conservation of amino acid residues in the enzyme family may provide information on the selection of target sites. Both conserved and nonconservedsites should be considered.

The sites responsible for catalytic mechanisms may be highly conserved or non-conserved. For example, in astudy by Mandelli et al., the distal residue Tyr195 was screened by sequence alignments of CrMan26 with other GH26 endo-β-1,4-mannanases because it was not strictly conserved; other residues found in the same position were Val, Gln, or Asp (Mandelli et al., 2020). In contrast, some non-active sites were chosen because of their high conservation (Gao et al., 2017; Joiner and Levine, 2019). For example, inthe meso-diaminopimelate dehydrogenase (*meso*-DAPDH) family, the non-active site was Ala69 in type I meso-DAPDHand was Arg71 in type II*meso*-DAPDH (Gao et al., 2017), both of which were highly conserved during the evolution of the respective types. Gao et al. created variants of R71A with the representative member of type II from*Symbiobacterium thermophilum* IAM14863 (StDAPDH) as a template to understand the mechanisms underlyingthe difference in substrate specificities between type I and type II meso-DAPDH. The results indicated that Arg71 in StDAPDH is an indicator of the amination preference of type II*meso*-DAPDH (Gao et al., 2017). Further analysis indicated that Arg71 maintained the catalytic conformations of Tyr205 by cation-π interactionsand interacted with the key catalytic residue His154 by hydrogen bonding (Zhang et al., 2018).

For improved enzymatic characterization, non-active sites that were not conserved were selected because of their greater tolerance toward mutations. However, this does not mean that conserved non-active sites cannot be used as targets to improve the catalytic performance of enzymes. For example, during the evolution of the DGD, Taylor et al. found that Ser306 was located near the DGD active sites and was highly conserved in DGD. The mutation S306F resulted in a 6-fold decrease in K_M_ and theconservation ranking of Ser306 was at the 10th percentile, indicating its evolutionary importance (Taylor et al., 2015).

Structural superimpositionand public bioinformatics tools such as Hotspots (Pavelka et al., 2009), and CAVER (Stourac et al., 2019)may be used toanalyzethe impact of 3D structureson the mining of target non-active sitesin order to increase the catalytic efficiency of endo-polygalacturonasePG8fn. Tu et al. substituted the non-active site Thr113 (Tu et al., 2016) based on the following considerations: 1) by multiple sequence alignment, Thr113 was found to be a distinct residue compared to other endo-polygalacturonases, which contained Gly at this position; 2) by structural superimposition, Thr113 was found to be located in the T3 loop, which existed in all of the proteins; and 3) by void pathway analysis byCAVER, Thr113 was found to be near the entrance of the T1 pathway.

Analysis of structural dynamics by molecular dynamic (MD) simulations can provide information on site selection. For example, docking, CAVER tools, and molecular dynamics (MD) simulations were employed together to choose the target residues for engineering CgTD for bulky substrates (Song et al., 2020). First, a docking analysis was performed to obtain the complex structures of CgTD with natural or unnatural bulk substrates. It was found that CgTD can accommodate bulky substrates according to volumetric estimates. Second, CAVER tools were employed to analyze the access tunnel, which indicated that the tunnels comprising bulky and hydrophobic residues formed a bottleneck structure for the entrance of the substrate. Finally, MD simulations were performed to determine how the substrates passed through the tunnel. The results showed that the tunnelwas gated with dynamic “open-closed” movements. According to the above analysis, the “open-gate” strategy was employed with the tunnel residues as targets.

In addition, knowledge ofthe relationship between the function and structure of enzymes is necessary. For example, His192 in exo-inulinase from *Aspergillus niger* 5,012 is a non-active site, which either lacks direct interaction with the substrate or is involved in the catalytic activity. Arjomandetal.mutated His192to Ala to investigate its role in the activity and specificity of exo-inulinase (Arjomand et al., 2017). The reasons were as follows: first, the review of histidine revealed that the imidazole ring of histidine can be involved in π-stacking interactions; second, the physiological pH of histidine made it flexible to be protonated positive or in a deprotonated neutral state of charge, and His192 is located at the end of a short connecting structure of the catalytic domain of exo-inulinase.

### Combined Analysis of Structural Dynamics and Evolutionary Analysis

Crystallographic structures, MD simulations, and normal mode analysis are popular bioinformaticapproaches for studying structural dynamics. However, these analyses are typically employed separately. For large protein families, systematic studies that integrate structural dynamics and evolutionary analysis may be a powerful tool for understanding enzymatic mechanisms (Skjærven et al., 2014). This correlation network analysis can be realized using the Bio3D package based on molecular sequences, crystallographic structural ensembles, and MD trajectories (Grant et al., 2006; Skjærven et al., 2014). For example, dynamic cross-correlation matrices (DCCMs) were generated by the analysis of Bio3D forWT and 3M variants of transketolase. According to the DCCMs, the residues His192, Ala282, Ile365, and Gly506 located in the dimer-interface regions were found to be correlated with the flexible regions within the active sites. Therefore, the four residues were introduced into variant 3M to improve their stability (Yu and Dalby, 2018a).

## Conclusion and Perspectives

In this mini-review, recent progress in research on non-active sites of enzymes has been summarized. Non-active sites of enzymes have received attention from researchers, regardless of the biocatalytic mechanism or enzyme engineering. However, withincreasing number of enzymes being discovered, some novel enzyme families with different functions are repressed, and similarto the *meso-DAPDH* family that is being researched by our group, questions are being raised about ways to engineer enzymatic performance with a guarantee of D-stereo-specificity.Questions also exist about why subfamilies exhibit distinctly different specificities under situations in which almost all of their active sites are the same. Non-active sites may be an effective beginning to the process of answering these questions. Therefore, in our opinion, non-active sites require extensive and in-depth studies.

According to the published results of enzyme engineering by directed evolution, variants with improved characterization contain non-active site mutations. However, for non-active sites, it is necessary to select possible residues for site-directed mutagenesis. In this review, we listed the locations of effective non-active sites and bioinformatic tools for mining the possible non-active sites. Although there are no direct interactions between non-active sites and the substrate, itwas noted that both conserved and non-conserved amino acid residues differ within an enzyme family, whichaffect the conformation of enzymes with respect to both the catalytic cavity and tunnel, and should bethe focusof future research. In the future, increasing the combinational employment of bioinformatictools based on amino acid sequences and 3D structures may greatly accelerate the progress of research on non-active sites. This will enrich the knowledge on the functional and structural relationships of enzymesand provide information on enzyme engineering.
